# Biopsychosocial Factors Associated With Return to Preinjury Sport After ACL Injury Treated Without Reconstruction: NACOX Cohort Study 12-Month Follow-up

**DOI:** 10.1177/19417381221094780

**Published:** 2022-05-27

**Authors:** Diane Slater, Joanna Kvist, Clare L. Ardern

**Affiliations:** † Unit of Physiotherapy, Department of Health, Medicine and Caring Science, Linköping University, Linköping, Sweden; ‡ Center for Medical Image Science and Visualization (CMIV), Department of Health, Medicine and Caring Sciences, Linköping University, Linköping, Sweden; § Stockholm Sports Trauma Research Center, Department of Molecular Medicine and Surgery, Karolinska Institute, Stockholm, Sweden; ‖ Sport and Exercise Medicine Research Centre, La Trobe University, Melbourne, Australia; ¶ Department of Family Practice, University of British Columbia, Vancouver, Canada

**Keywords:** knee, prognosis, rehabilitation, return to sport, sports medicine

## Abstract

**Background:**

The limited research on prognosis after nonsurgical management of anterior cruciate ligament (ACL) injury has focused on physical factors. We aimed to assess relationships between key patient-reported outcomes, in line with a biopsychosocial approach, and returning to preinjury sport at 12 months after ACL injury treated without reconstruction.

**Hypothesis:**

We hypothesized that biopsychosocial factors would be associated with returning to preinjury sport at 12 months after ACL injury.

**Study Design:**

Prospective single cohort study.

**Level of Evidence:**

Level 2.

**Methods:**

Patients who had an ACL injury and did not have reconstruction during the first year after injury were recruited from healthcare clinics in Sweden, and followed up at 3, 6, and 12 months after injury. Return to preinjury sport at 12 months was the primary outcome. Explanatory variables were psychological readiness to return to sport, knee-related quality of life, and self-reported knee function. Using generalized estimating equations, we evaluated the relationships between the explanatory variables and the primary outcome at each timepoint.

**Results:**

Data were analyzed for 88 participants with a median age of 27 years (15-40 years). Soccer was the most frequently reported preinjury sport (n = 22). Forty participants (46%) had returned to their preinjury sport at 12 months after ACL injury. The odds of returning to preinjury sport at 12 months increased with higher self-reported knee function at 6 months (odds ratio [OR], 1.1; 95% CI, 1.0-1.1), and the odds of being returned to the preinjury sport at 12 months doubled for every 1-point increase (1-10 scale) in psychological readiness to return to sport measured at 12 months (OR, 1.9; 95% CI, 1.2-3.2).

**Conclusion:**

Superior self-reported knee function at 6 months and greater psychological readiness to return to sport at 12 months were associated with returning to the preinjury sport 1 year after ACL injury treated without reconstruction.

**Clinical Relevance:**

Consider highlighting the relevance of biopsychosocial factors to returning to preinjury sport after ACL injury when discussing prognosis during shared decision-making.

Returning to preinjury sport is important to patients after anterior cruciate ligament (ACL) injury, and the desire to return influences treatment choice.^6,11,13^ Nonsurgical and surgical management are acceptable primary treatment options for ACL injury.^6,27^ During shared decision-making for treatment, patients need to be informed about the evidence for return to sport associated with different ACL injury treatment options.^6^ This discussion should include expected return-to-sport rates and factors that could affect returning to sport. Research regarding return to sport after ACL injury is weighted heavily toward prognosis after ACL reconstruction. Understanding more about prognostic factors for return to sport after nonsurgical management of ACL injury will help clinicians and patients make informed decisions about treatment.

The patient attempting to return to their preinjury sport after ACL injury, irrespective of nonsurgical or surgical treatment, faces many challenges during the prolonged rehabilitation period and at the time of transition back to sport. Patients are exposed to different biological, psychological, and social factors. The biopsychosocial model (adapted to sports injury rehabilitation) helps clinicians and researchers understand which factors related to the individual may interact to influence outcomes after sports injury.^8,26^

The limited research on prognosis after nonsurgical management of ACL injury has focused on physical factors.^7,16,37^ Research on prognosis after ACL reconstruction has shown that sufficient knee function does not guarantee returning to sport.^4^ Psychological factors, including confidence, self-efficacy, fear of reinjury, stress, and motivation influence return to sport after ACL reconstruction.^2,9,31^ Fear of reinjury is the most common reason why athletes do not return to their preinjury sport after surgery.^3^ Other factors that influence return to preinjury sport after ACL reconstruction include the social support available to the injured athlete, preinjury sports level, risk taking behaviors, adherence to rehabilitation, job demands, and family commitments.^9,31,32^ It is possible, but not previously studied, that similar factors also affect returning to sport after nonsurgical management of ACL injury. The interplay between these factors is also unclear.

Using a biopsychosocial approach, we aimed to assess relationships between key patient-reported outcomes, measured at clinically relevant time points, and returning to the preinjury sport at 12 months after an ACL injury treated without reconstructive surgery.

## Methods

### Design and Participants

The NACOX study is a multicenter prospective cohort study of the natural corollaries and recovery of patients with an acute ACL injury. It was approved by the ethical authority in Linköping, Sweden (Dnr 2016/44-31 and 2017/221-32) and prospectively registered (ClinicalTrials.gov

identifier: NCT02931084).^19^ Data analyses for this study were preplanned. Funding organizations had no role in study design, data collection and analysis, decision to publish, or preparation of the manuscript.

Patients were recruited from public and private healthcare clinics in Sweden, reflecting typical clinical management of ACL injury in Sweden. Patients were recruited between October 2016 and October 2018 and provided informed consent to participate (see Appendix A for detail of procedures for recruitment, consent and online data collection).

Patients with a clinical diagnosis of ACL rupture from an orthopaedic surgeon, verified by magnetic resonance imaging, no more than 6 weeks before recruitment, and who were aged between 15 and 40 years (inclusive) at the time of injury were included in the NACOX study. The exclusion criteria were as follows: serious concomitant knee injury (eg, fracture requiring separate treatment, posterior cruciate ligament rupture), previous ACL injury on the same knee, inability to understand spoken and written Swedish, cognitive impairments, and other injury or illness or chronic pain diagnosis associated with impaired function (eg, rheumatic disease, fibromyalgia).

We analyzed data from a single cohort of patients who had nonsurgical management during the first year after their ACL injury. Patients were excluded if they did not respond at the 12-month follow-up, or if they had an arthroscopy to their injured knee within 3 months before completing the 12-month follow-up questionnaire, or if they did not provide any return-to-sport data at 12 months. Data from participants who had an arthroscopy 14 days or fewer before completing the 3- or 6-month questionnaires were excluded from the analysis at that timepoint.

### Measures and Outcomes

The primary outcome was return to preinjury sport at 12 months after ACL injury. We used the question: “Have you returned to your preinjury sport?” (answer options: yes/no). Participants who had not returned to their preinjury sport were asked their reason(s) for not returning, and their sport and physical activity participation goals. If there were missing data for the primary outcome, reasons for not returning were reviewed. For participants who did not answer this question, information from routine physical activity surveillance conducted as part of the NACOX study (physical activity participation data collected every month by electronic questionnaire) was substituted.^19^ We excluded participants if no information was available for substitution.

#### Explanatory Variables

We selected the following 3 patient-reported outcome measures to evaluate a range of biological, psychological, and social factors. Each measure has evidence of adequate measurement properties for evaluating outcomes in patients with ACL injury.^5,20,21^

- The Swedish version of the International Knee Documentation Committee (IKDC) Subjective Knee Form (IKDC-SKF) to assess self-reported knee function at 3, 6, and 12 months after ACL injury.^12^
- The Swedish version of the ACL-Return to Sport after Injury (ACL-RSI) (1-10) scale to evaluate psychological readiness to return to sport at 3, 6, and 12 months after ACL injury.^35^

- The Swedish version of the ACL-Quality of Life (ACL-QOL) questionnaire to assess condition-specific quality of life at 3 months and 12 months after ACL injury. To minimize participant burden, the ACL-QOL was not collected at the 6-month follow-up.

#### Baseline Measures

We used the following 3 baseline measures:

- Preinjury sport classified using the modified Tegner Activity Scale^30^ and the IKDC sports activity level classification.^18,25^
- The Swedish version of the General Self-Efficacy Scale (GSES).^22^
- The Single Assessment Numeric Evaluation (SANE) rating for global knee function.^28^

See Appendix B for additional information relating to patient-reported outcome measures.

### Statistical Analysis

Data were analyzed with IBM SPSS Statistics for MacIntosh, version 25.0 (IBM Corp) and Stata Statistical Software, version 16 (StataCorp).

Descriptive statistics were calculated for baseline variables and for explanatory variables at 3 months, 6 months, and 12 months after ACL injury. We completed univariable analyses of the between-group differences in baseline data and of the between-group differences in explanatory variables at 3, 6, and 12-month follow-up. A *P* value of <0.05 denoted statistical significance.

#### Multivariable Analysis

We used generalized estimating equations (GEE) (binomial distribution, logit link function) to examine the association between return to preinjury sport at 12 months and the explanatory variables. We constructed 3 models to evaluate the relationships between the explanatory variables and the primary outcome at each time point: 3, 6, and 12 months. To ensure we retained sufficient power and adequate model performance, in line with published recommendations, we estimated the target number of candidate explanatory variables before selecting the variables.^29^ Age at ACL injury, GSES, and preinjury Tegner Activity Scale score were included as adjusting variables in each model (see Appendix C for details of the univariable and multivariable analysis including protocols relating to missing data and risk of response bias).

## Results

### Demographic and Baseline Factors

**Figure 1.**
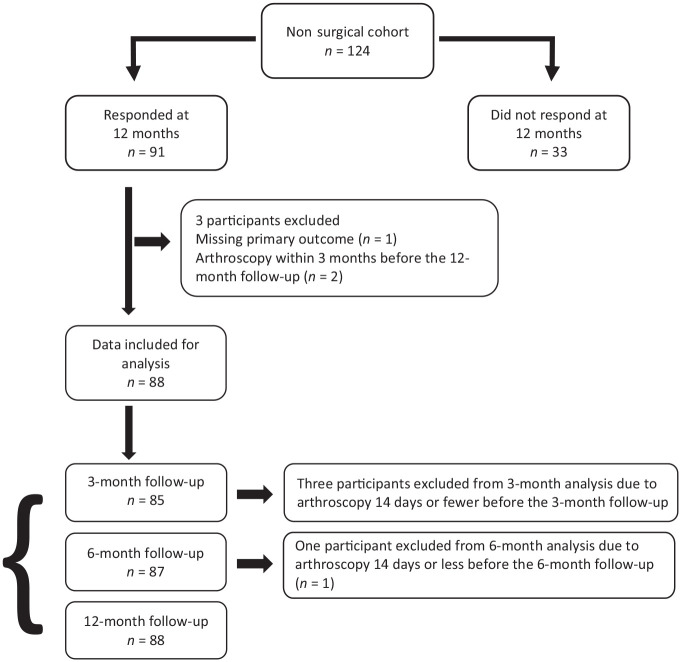
Flow of participants through the study; *n*, number of participants.

A total of 124 patients had not had ACL reconstruction at 1 year after injury and 91 patients (47 men and 44 women) met the inclusion criteria (Figure 1). Demographic and baseline characteristics for the 88 participants included in data analysis are shown in Table 1. There was no significant difference in age, sex, preinjury activity level, or baseline SANE between those who responded to the 12-month follow-up and those who did not (n = 33). Two-thirds of the participants were employed, and one-third were students. Nearly half of participants reported participating in competitive sports before their ACL injury and the most frequently reported preinjury sport was soccer (n = 22) (Appendix D). There was a statistically significant difference in the proportion distributions of preinjury sport classified according to the modified IKDC activity level (χ^2^ = 14.391; *P* < 0.01) for those who had returned to preinjury sport and those who had not. For level II preinjury sports, more participants had not returned than had returned, and for level III preinjury sports more participants had returned than had not returned (Table 1). The 40 participants who had returned to their preinjury sport at 12 months were older than participants who had not returned (*U* = 1282, *z* = 2.698, *P* = 0.01).

**Table 1.** Demographic and baseline data for participants who returned and did not return to preinjury sport at 12 months after ACL injury treated without reconstruction

		Returned to Preinjury Sport		
Baseline Variables	Total , n = 88	Yes, n = 40	No, n = 48	*P*
Age; median (IQR), years	27 (11.4)	28 (11.3)	24 (10.2)	0.01^*a*^
Sex; n				
Male	44 (50%)	22 (55%)	22 (46%)	0.39^*b*^
Female	44 (50%)	18 (45%)	26 (54%)	
Concomitant injury at time of index injury; n^*c*^				
Collateral ligament Intact/partial tear	60 (70%)	27 (68%)	33 (72%)	0.67^*b*^
Complete rupture	26 (30%)	13 (32%)	13 (28%)	
Meniscus Intact/partial tear	56 (65%)	28 (70%)	28 (61%)	0.38^*b*^
Complete rupture	30 (35%)	12 (30%)	18 (39%)	
Cartilage Intact	80 (93%)	38 (95%)	42 (91%)	0.68^*d*^
Injury	6 (7%)	2 (5%)	4 (9%)	
Days from injury to 12-month follow-up; Median (IQR)	369 (13)	368 (14)^*e*^	369 (15)^*f*^	0.39^*a*^
Preinjury Tegner Activity Scale; Median (IQR), level	6.5 (5)	4 (5)	7 (5)	0.14^*a*^
Preinjury IKDC activity level; n				
Level I	39 (44%)	16 (40%)	23 (48%)	<0.01^*b*^
Level II	14 (16%)	1 (3%)	13 (27%)^*g*^	
Level III	35 (40%)	23 (57%)	12 (25%)^*g*^	
General self-efficacy scale; mean (SD)	32 (3.7)^*h*^	33 (3.5)^*h*^	32 (4.0)	0.73^*i*^
SANE rating; mean (SD)	40 (20.1)^j,k^	42 (21.0)^*k*^	39 (19.4)^*j*^	0.49^*i*^

ACL, anterior cruciate ligament; IKDC, International Knee Documentation Committee; IQR, interquartile range; SANE, Single Assessment Numeric Evaluation.

a Mann-Whitney *U* test asymptotic *P* value.

b Chi-square test of homogeneity (all expected cell counts were ≥5).

c Radiological data not available for 2 participants who had not returned to preinjury sport at 12 months (% of 86 for total group, % of 46 for not returned group).

d Fisher’s exact test.

e Five outliers (399, 408, 408, 414, 419 days from injury) not excluded from analysis.

f Four outliers (341, 400, 400, 412 days from injury) not excluded from analysis.

g Multiple *Z* test *P* < 0.02.

h Data missing from 1 participant.

i Independent samples *t* tests.

j Data missing from 1 participant.

k One outlier (SANE 85) not excluded from analysis.

### Biopsychosocial Factors

Participants who had returned to their preinjury sport at 12 months reported superior knee function (ie, higher IKDC-SKF) at 6 and 12 months, superior knee-related quality of life (ie, higher ACL-QOL) at 3 and 12 months, and superior psychological readiness to return to sport (ie, higher ACL-RSI) at 3, 6, and 12 months compared with participants who had not returned (Table 2).

**Table 2.** Biopsychosocial factors measured at 3, 6, and 12 months after ACL injury for participants who returned and did not return to preinjury sport at 12 months after ACL injury treated without reconstruction (univariable analysis)

		Returned to Preinjury Sport at 12 Months				
Explanatory Variables	Total	Yes	No	*t* (df)	Mean Difference (95% CI)	*P* ^*a*^
Measured at 3 months after ACL injury						
IKDC-SKF mean (SD)	59.0 (15.4) n = 76^*b*^	62.7 (14.5) n = 35	55.8 (15.7) n = 41^*b*^	2.0 (74)	7.0 (0.0-13.9)	0.05
ACL-QOL mean (SD)	5.3 (1.7) n = 72^*b*^	5.8 (1.7) n = 34	4.8 (1.7) n = 38^*b*^	2.3 (70)	0.9 (0.1-1.7)	0.03
ACL-RSI mean (SD)	4.6 (2.0) n = 71^*b*^	5.2 (2.1) n = 33	4.0 (1.8) n = 38^*b*^	2.6 (69)	1.2 (0.3-2.1)	0.01
Measured at 6 months after ACL injury						
IKDC-SKF mean (SD)	70.5 (14.8) n = 78^*c*^	75.9 (13.7) n = 36^*c*^	65.9 (14.3) n = 42	3.1 (76)	10.0 (3.7-16.3)	<0.01
ACL-RSI mean (SD)	5.1 (2.1) n = 76^*c*^	5.9 (2.2) n = 35^*c*^	4.5 (1.7)^*d*^ n = 41	3.3 (74)	1.5 (0.6-2.4)	<0.01
Measured at 12 months after ACL injury						
IKDC-SKF mean (SD)	75.2 (15.3)^*e*^ n = 86	81.6 (11.7) n = 39	70.0 (16.0) n = 47	3.9 (83)	11.6 (5.7-17.5)	<0.01f
ACL-QOL mean (SD)	6.8 (1.9) n = 84	7.8 (1.6) n = 38	5.9 (1.7) n = 46	5.3 (82)	1.9 (1.2-2.6)	<0.01
ACL-RSI mean (SD)	5.7 (2.5) n = 84	7.2 (2.1) n = 39	4.4 (2.0) n = 45	6.1 (82)	2.8 (1.9-3.7)	<0.01

ACL, anterior cruciate ligament; ACL-QOL, ACL knee-related quality of life (range 1-10); ACL-RSI, ACL psychological readiness (range 1-10); IKDC, International Knee Documentation Committee; IKDC-SKF, IKDC subjective knee function (range 0-100); *n*, number of participants with data for explanatory variables at the relevant time point.

a Independent samples *t* tests unless otherwise stated.

b Responses excluded for 3 participants due to arthroscopy.

c Responses excluded for 1 participant due to arthroscopy.

d One outlier (ACL-RSI 8.8) not excluded from analysis.

e One outlier (IKDC-SKF 34.5) not excluded from analysis.

f Welch *t*-test.

From the multivariable analysis, when accounting for age, preinjury Tegner, and GSES, biopsychosocial factors measured at 3 months after ACL injury did not predict returning to preinjury sport at 12 months (Table 3). For every 1 point increase in IKDC-SKF score measured at 6 months, the odds of returning to the preinjury sport at 12 months increased by 10%. For every 1 point increase in ACL-RSI score (1-10 scale) measured at 12 months, the odds of being returned to the preinjury sport at 12 months approximately doubled (Table 3) (see Appendix E for missing data summary; see Appendix F and G for unadjusted GEE models).

**Table 3.** Biopsychosocial factors at 3 months, 6 months, and 12 months, for participants who returned and did not return to preinjury sport at 12 months after ACL injury treated without reconstruction (multivariable analysis, adjusted data)

	GEE Model 1: 3 Months^*a*^ (n = 71)		GEE Model 2: 6 Months^*b*^ (n = 76)		GEE Model 3: 12 Months^*c*^ (n = 82)	
Explanatory Variables	OR (95% CI)	*P*	OR (95% CI)	*P*	OR (95% CI)	*P*
IKDC-SKF	1.0 (1.0-1.1)	0.61	1.1 (1.0-1.1)	0.02	1.1 (1.0-1.1)	0.16
ACL-QOL^*d*^	1.1 (0.6-2.0)	0.85	-	-	0.8 (0.4-1.6)	0.49
ACL-RSI	1.3 (0.8-2.1)	0.28	1.1 (0.8-1.6)	0.52	1.9 (1.2-3.2)	0.01
Adjusting Variables						
Age	1.1 (1.0-1.2)	0.03	1.1 (1.0-1.2)	0.01	1.1 (1.0-1.2)	0.03
Preinjury Tegner Activity Scale	1.0 (0.8-1.3)	0.76	1.0 (0.8-1.2)	0.63	1.0 (0.8-1.3)	0.97
GSES baseline	1.0 (0.8-1.1)	0.55	1.0 (0.9-1.2)	0.96	1.0 (0.8-1.1)	0.62

ACL, anterior cruciate ligament; ACL-QOL, ACL knee-related quality of life (range, 1-10); ACL-RSI, ACL psychological readiness (range, 1-10); GSES, General Self Efficacy scale; IKDC, International Knee Documentation Committee; IKDC-SKF, IKDC subjective knee function (range, 0-100); OR, odds ratio.

a Goodness of fit QIC 99.320/ QICC 99.023.

b Goodness of fit QIC 94.994/ QICC 95.111.

c Goodness of fit QIC 90.494/ QICC 91.071.

d The ACL-QOL was not included in the 6-month follow-up questionnaire.

### Reasons for Not Returning to Preinjury Sport

Fear of reinjury and poor knee function were the most frequent reasons for not returning to preinjury sport (Table 4). At 12 months, 12 participants (25% of 48 who had not returned to preinjury sport) no longer had a goal to return to preinjury sport (Table 5).

**Table 4.** Reasons for not returning to preinjury sport at 12-month follow-up

Reasons for not Returning to Preinjury Sport	Number of Participants, n (% of 48)
Fear of reinjury	15 (31)
Poor knee function	11 (23)
Undergoing rehabilitation	9 (19)
Lack of confidence in knee	7 (15)
Other	6 (12)
Not specified	1
All of the above	1
Participating in a different sport	2
Postpartum	1
Not participating in any sport	1

**Table 5.** Goals related to returning to sport at baseline and at 12-month follow-up for those who had not returned to their preinjury sport 12 months after ACL injury

Goal Relating to Return to Sport	Baseline, n (% of 47)^*a*^	12-Month Follow-up, n (% of 48)
Return to preinjury sport	45 (96)	34 (71)
Return to a different sport	2 (4)	12 (25)
Not to return to sport	0 (0)	2 (4)

a Data missing for 1 participant. ACL, anterior cruciate ligament.

## Discussion

Individual biological, psychological, and social factors reported at 3, 6, and 12 months after injury were relevant to returning to preinjury sport at 12 months after injury. However, when adjusted for age, preinjury activity level and general self-efficacy, their influence was attenuated. In our multivariable model, returning to preinjury sport was predicted by superior self-reported knee function at 6 months, associated with greater psychological readiness at 12 months, and not associated with knee-related quality of life at any of the timepoints. It is possible for patients who do not have ACL reconstruction to return to their preinjury sport, including pivoting sports, 12 months after an ACL injury. However, many patients (54%) do not achieve this outcome. Reasons for not returning at 12 months covered physical, social, and psychological domains.

### Evaluating Biopsychosocial Factors in Sports Medicine Clinical Practice

After sustaining an ACL injury, patients want to know whether, and when, they will be able to return to their preinjury sport. Our results suggest that evaluating biopsychosocial factors early in rehabilitation (at 3 months) may not help clinicians make prognoses about return to preinjury sport. In previous research, self-reported knee function at approximately 3 months predicted successful outcome, but a successful outcome was not defined as return to sport.^16^ Early measurements of psychological readiness to return to sport, before surgery and at 4 months after ACL reconstruction, predicted returning to preinjury sport at 12 months.^1^ It is unclear why early measurement of psychological readiness to return to sport predicted return to preinjury sport for patients with ACL reconstruction, but not for patients with nonsurgical treatment in our study.

Superior self-reported knee function at 6 months may indicate that the athlete is on track to be physically ready to return to sport; lower IKDC-SKF scores may help identify patients who require modifications to their rehabilitation. It is unclear why we did not find a relationship between self-reported knee function at 12 months and returning to sport, considering 4 in every 10 participants who had not returned to their preinjury sport at 12 months reported either poor knee function or ongoing rehabilitation as the reason. Our results might reflect that some patients return to sport despite having poor knee function.

Consistent with findings from ACL reconstruction cohorts,^1,17,36^ psychological readiness to return to sport, measured with the ACL-RSI, was associated with returning to sport at 12 months in people who received nonsurgical treatment. Recent consensus from experts in managing ACL injury is that clinicians should assess psychological readiness in the last phase of rehabilitation, as part of the return to sport decision-making process.^6^ However, when interpreting the ACL-RSI scores at 12 months, when some participants have already returned to their preinjury sport, it is uncertain whether a positive psychological response causes return to sport or whether returning to sport causes a positive psychological response.

The different relationships at 6 and 12 months between return to sport and subjective knee function, and return to sport and psychological readiness, may be explained by the dynamic nature of biopsychosocial factors. Clinicians should be aware of the limitations of questionnaires and consider the impact of timing on questionnaire responses. Clinicians may choose to use a range of patient-reported outcome measures to monitor progress during rehabilitation, but not to make predictions for return to sport after ACL injury.

### Informed Treatment Decision-Making and Managing Patient Expectations

The shared decision-making approach is an opportunity to help patients establish realistic expectations about returning to sport. Return to preinjury sport was a goal held by the overwhelming majority of participants at baseline (96%), yet many did not achieve it. The 46% return to preinjury sport rate in our study is lower than previously published 12-month rates for a nonsurgical cohort in Norway (65-69%).^15,24,25^ The low return rate may be due to differences in rehabilitation. Our study reflects usual care delivered across Sweden compared with the specialist care received by participants in the Norwegian cohort. A similar range of return to preinjury sport rates has been reported in ACL reconstruction cohorts, and this also seems to reflect specialist care versus “usual care.”^1,15^

The 12-month follow-up for return to preinjury sport is clinically relevant. In ACL reconstruction cohorts, patients often expect to return to sport within the year after their ACL injury.^10^ In our study, some participants were undergoing rehabilitation at 12 months, which may reflect the presence of ongoing or new knee problems. Long-term follow-up would provide information regarding delayed decisions to modify activity or undergo ACL reconstruction.

Almost half of the participants in our study participated in level I sports before injury, and 2 in every 5 had returned to their preinjury sport at 12 months without having an ACL reconstruction, which is similar to previous research.^14,15^ Current recommendations for patients who wish to return to jumping, cutting, and pivoting sports is early ACL reconstruction after a period of preoperative rehabilitation.^6,11^ The rationale behind the recommendation is that surgical reconstruction or activity modification is necessary to avoid subsequent meniscal and cartilage injuries, which may contribute to posttraumatic osteoarthritis.^6,34^ We evaluated the relationship between relevant biopsychosocial factors that can be monitored during rehabilitation, and return to preinjury sport. We did not seek to answer the question “should patients return to level I sport without reconstructive surgery?” Follow-up data of new injuries and ongoing sports participation may provide some answers to that question.

The reasons for not returning to preinjury sport varied. Knee-related psychological factors (fear of reinjury and a lack of knee-related confidence) were the most common reasons for not returning to the preinjury sport at 12 months, which support previous research in patients with ACL reconstruction.^2^ Almost one-third of the participants who had not returned to preinjury sport at 12 months had changed their return-to-sport goal from baseline: they no longer wanted to return to their preinjury sport.

### Strengths and Limitations

The biopsychosocial model is complex, and factors additional to those measured in our study are also likely to be relevant to returning to sport. Larger studies are required for multivariable analyses that include a larger number of biopsychosocial variables.

Mixed methods approaches may be better suited to evaluating social factors, given they are highly individual.^33^ We used the ACL-QOL questionnaire as an indirect measure of social factors, as it includes questions covering work-related, lifestyle, social and emotional concerns.^23^ In our multivariable model, the ACL-QOL score was not associated with return to preinjury sport at any time point. Qualitative research in ACL reconstruction cohorts has identified that social factors, including job demands, life priorities, and family commitments influence return to sport.^32^

The heterogenous population is a good representation of patients in Sweden who have nonsurgical management after an ACL injury. Adjusting for age, preinjury activity level, and self-efficacy improved the generalizability of our results. However, our results may not generalize to countries where approaches to managing ACL injuries are very different to those in Sweden.

## Conclusion

Superior self-reported knee function at 6 months and greater psychological readiness to return to sport at 12 months were associated with returning to the preinjury sport 1 year after ACL injury treated without reconstructive surgery. More than half of participants had not returned to their preinjury sport by 1 year after nonsurgical management of ACL injury.

## Clinical Relevance

The data support the following:

- Biopsychosocial factors are relevant to returning to preinjury sport at 12 months after ACL injury for those who have nonsurgical management. We suggest clinicians consider highlighting the role of biopsychosocial factors when discussing prognosis during the shared decision-making process.
- It may be appropriate for clinicians to use a range of patient-reported outcome measures to monitor progress during rehabilitation, but not to make predictions for return to sport. Clinicians should be aware of the limitations of questionnaires and consider the impact of timing on questionnaire responses.
- At 1 year after ACL injury treated with nonsurgical management, many patients had not achieved their goal of returning to preinjury sport. It may be important to manage patient expectations.

## Supplemental Material

sj-docx-1-sph-10.1177_19417381221094780 – Supplemental material for Biopsychosocial Factors Associated With Return to Preinjury Sport After ACL Injury Treated Without Reconstruction: NACOX Cohort Study 12-Month Follow-up Click here for additional data file. Supplemental material, sj-docx-1-sph-10.1177_19417381221094780 for Biopsychosocial Factors Associated With Return to Preinjury Sport After ACL Injury Treated Without Reconstruction: NACOX Cohort Study 12-Month Follow-up by Diane Slater, Joanna Kvist and Clare L. Ardern in Sports Health: A Multidisciplinary Approach

sj-docx-2-sph-10.1177_19417381221094780 – Supplemental material for Biopsychosocial Factors Associated With Return to Preinjury Sport After ACL Injury Treated Without Reconstruction: NACOX Cohort Study 12-Month Follow-up Click here for additional data file. Supplemental material, sj-docx-2-sph-10.1177_19417381221094780 for Biopsychosocial Factors Associated With Return to Preinjury Sport After ACL Injury Treated Without Reconstruction: NACOX Cohort Study 12-Month Follow-up by Diane Slater, Joanna Kvist and Clare L. Ardern in Sports Health: A Multidisciplinary Approach

sj-docx-3-sph-10.1177_19417381221094780 – Supplemental material for Biopsychosocial Factors Associated With Return to Preinjury Sport After ACL Injury Treated Without Reconstruction: NACOX Cohort Study 12-Month Follow-up Click here for additional data file. Supplemental material, sj-docx-3-sph-10.1177_19417381221094780 for Biopsychosocial Factors Associated With Return to Preinjury Sport After ACL Injury Treated Without Reconstruction: NACOX Cohort Study 12-Month Follow-up by Diane Slater, Joanna Kvist and Clare L. Ardern in Sports Health: A Multidisciplinary Approach

sj-docx-4-sph-10.1177_19417381221094780 – Supplemental material for Biopsychosocial Factors Associated With Return to Preinjury Sport After ACL Injury Treated Without Reconstruction: NACOX Cohort Study 12-Month Follow-up Click here for additional data file. Supplemental material, sj-docx-4-sph-10.1177_19417381221094780 for Biopsychosocial Factors Associated With Return to Preinjury Sport After ACL Injury Treated Without Reconstruction: NACOX Cohort Study 12-Month Follow-up by Diane Slater, Joanna Kvist and Clare L. Ardern in Sports Health: A Multidisciplinary Approach

sj-docx-5-sph-10.1177_19417381221094780 – Supplemental material for Biopsychosocial Factors Associated With Return to Preinjury Sport After ACL Injury Treated Without Reconstruction: NACOX Cohort Study 12-Month Follow-up Click here for additional data file. Supplemental material, sj-docx-5-sph-10.1177_19417381221094780 for Biopsychosocial Factors Associated With Return to Preinjury Sport After ACL Injury Treated Without Reconstruction: NACOX Cohort Study 12-Month Follow-up by Diane Slater, Joanna Kvist and Clare L. Ardern in Sports Health: A Multidisciplinary Approach

sj-docx-6-sph-10.1177_19417381221094780 – Supplemental material for Biopsychosocial Factors Associated With Return to Preinjury Sport After ACL Injury Treated Without Reconstruction: NACOX Cohort Study 12-Month Follow-up Click here for additional data file. Supplemental material, sj-docx-6-sph-10.1177_19417381221094780 for Biopsychosocial Factors Associated With Return to Preinjury Sport After ACL Injury Treated Without Reconstruction: NACOX Cohort Study 12-Month Follow-up by Diane Slater, Joanna Kvist and Clare L. Ardern in Sports Health: A Multidisciplinary Approach

sj-docx-7-sph-10.1177_19417381221094780 – Supplemental material for Biopsychosocial Factors Associated With Return to Preinjury Sport After ACL Injury Treated Without Reconstruction: NACOX Cohort Study 12-Month Follow-up Click here for additional data file. Supplemental material, sj-docx-7-sph-10.1177_19417381221094780 for Biopsychosocial Factors Associated With Return to Preinjury Sport After ACL Injury Treated Without Reconstruction: NACOX Cohort Study 12-Month Follow-up by Diane Slater, Joanna Kvist and Clare L. Ardern in Sports Health: A Multidisciplinary Approach
