# Supplementary material for: Biopsychosocial Factors Associated With Return to Preinjury Sport After ACL Injury Treated Without Reconstruction: NACOX Cohort Study 12-Month Follow-up
Source: Sports Health. 2022 May 27;15(2):176–84. doi: 10.1177/19417381221094780 (PMC9950991; doi:10.1177/19417381221094780)
Supplement: sj-docx-1-sph-10.1177_19417381221094780 – Supplemental material for Biopsychosocial Factors Associated With Return to Preinjury Sport After ACL Injury Treated Without Reconstruction: NACOX Cohort Study 12-Month Follow-up [file sj-docx-1-sph-10.1177_19417381221094780.docx]

**Appendix A:** **Method procedures – recruitment, consent and online data collection**

We used an online questionnaire, constructed and administered using a web-based platform (Esmaker 3.0, Entergate AB), for all data collection. Participants were informed about the NACOX study at their initial contact with a healthcare provider following the ACL injury. A member of the research team contacted potential participants by telephone as soon as possible after the injury. This provided an opportunity for any questions related to the NACOX study to be answered and verbal consent to be obtained. Patients who accepted participation provided informed consent when they received the initial questionnaire. A uniform resource locator (URL) link to the initial and follow-up questionnaires were sent via short message service (SMS) or email to all patients who accepted participation. Up to three reminders were sent to participants who did not respond.

The baseline questionnaire collected demographic and preinjury characteristics including age, preinjury activity level and general self-efficacy. Participants completed the baseline questionnaire within 6 weeks of ACL injury. We collected follow-up data via follow-up questionnaires, sent at 3, 6 and 12 months following the ACL injury. Completion of each questionnaire took approximately 10 to 15 minutes.
