# Supplementary material for: Biopsychosocial Factors Associated With Return to Preinjury Sport After ACL Injury Treated Without Reconstruction: NACOX Cohort Study 12-Month Follow-up
Source: Sports Health. 2022 May 27;15(2):176–84. doi: 10.1177/19417381221094780 (PMC9950991; doi:10.1177/19417381221094780)
Supplement: sj-docx-2-sph-10.1177_19417381221094780 – Supplemental material for Biopsychosocial Factors Associated With Return to Preinjury Sport After ACL Injury Treated Without Reconstruction: NACOX Cohort Study 12-Month Follow-up [file sj-docx-2-sph-10.1177_19417381221094780.docx]

**Appendix B:** **Patient-reported outcome** **measures**

*Baseline measures*

Preinjury sport was classified using two separate systems: (1) modified Tegner Activity Scale^8^ which grades the sport activity (separate from work) with regard to knee demand and competition level, and (2) International Knee Documentation Committee (IKDC) sports activity level classification, which is based on the pivoting and contact demands of sports (level I, pivoting and contact sports like soccer and basketball; level II, pivoting and non-contact sports like tennis and alpine skiing; level III, sports with no pivoting and no contact like cycling, swimming and cross-country skiing).^2,6^

We used the Swedish version of the General Self Efficacy scale (GSES)^4^ to assess self-efficacy. A higher total score of the 10 items (maximum score: 40) indicates greater self-efficacy. We assessed global knee function at baseline using the single assessment numeric evaluation (SANE) rating; participants scored their knee from 0 to 100, with 100 being the best.^7^

*Explanatory variables*

We assessed self-reported knee function using the validated Swedish version of the International Knee Documentation Committee Subjective Knee Form (IKDC-SKF)^1^ at 3, 6 and 12 months following ACL injury. The IKDC-SKF comprises 18 items (mixture of dichotomous, 5-point Likert and 11-point rating scales), and a higher score indicates superior knee function.

We used the ACL-Return to Sport after Injury (ACL-RSI) scale,^9^ which comprises 12 questions on emotions, risk appraisal and confidence in performance, to evaluate psychological readiness to return to sport at 3, 6 and 12 months following ACL injury. In the culturally adapted, Swedish version, each question is answered on a 1-10-point Likert scale.^3^ A higher score indicates a more positive psychological response.

We used the ACL-Quality of Life (ACL-QOL) questionnaire to assess condition-specific quality of life at 3 months and 12 months following ACL injury. The original ACL-QOL^5^ is a 31-item questionnaire; the Swedish translation (with cross-cultural adaptation, and validation [unpublished data]) comprises 32 questions, answered on a 10-point Likert scale. A higher total score indicates superior knee-related quality of life. To minimise participant burden, the ACL-QOL was not collected at the 6-month follow-up.

References

1. Grevnerts HT, Grävare Silbernagel K, Sonesson S, et al. Translation and testing of measurement properties of the Swedish version of the IKDC subjective knee form. *Scand J Med Sci Sports*. 2017;27(5):554-562. doi:https://doi.org/10.1111/sms.12861

2. Hefti E, Müller W, Jakob RP, Stäubli H-U. Evaluation of knee ligament injuries with the IKDC form. *Knee Surg Sports Traumatol Arthrosc*. 1993;1(3-4):226-234. doi:https://doi.org/10.1007/BF01560215

3. Kvist J, Österberg A, Gauffin H, Tagesson S, Webster K, Ardern C. Translation and measurement properties of the Swedish version of ACL-Return to Sports after Injury questionnaire: Measurement properties ACL-RSI. *Scand J Med Sci Sports*. 2013;23(5):568-575. doi:https://doi.org/10.1111/j.1600-0838.2011.01438.x

4. Löve J, Moore CD, Hensing G. Validation of the Swedish translation of the general self-efficacy scale. *Qual Life Res*. 2012;21(7):1249-1253. doi:https://doi.org/10.1007/s11136-011-0030-5

5. Mohtadi N. Development and Validation of the Quality Of Life Outcome Measure (Questionnaire) for Chronic Anterior Cruciate Ligament Deficiency. *Am J Sports Med*. 1998;26(3):350-359. doi:https://doi.org/10.1177/03635465980260030201

6. Moksnes H, Snyder-Mackler L, Risberg MA. Individuals With an Anterior Cruciate Ligament-Deficient Knee Classified as Noncopers May Be Candidates for Nonsurgical Rehabilitation. *J Orthop Sports Phys Ther*. 2008;38(10):586-595. doi:https://doi.org/10.2519/jospt.2008.2750

7. Shelbourne KD, Barnes AF, Gray T. Correlation of a Single Assessment Numeric Evaluation (SANE) Rating With Modified Cincinnati Knee Rating System and IKDC Subjective Total Scores for Patients After ACL Reconstruction or Knee Arthroscopy. *Am J Sports Med*. 2012;40(11):2487-2491. doi:https://doi.org/10.1177/0363546512458576

8. Tegner Y, Lysholm J. Rating Systems in the Evaluation of Knee Ligament Injuries. *Clin Orthop*. 1985;Sept(198):43-49. doi:https://doi.org/10.1097/00003086-198509000-00007

9. Webster KE, Feller JA, Lambros C. Development and preliminary validation of a scale to measure the psychological impact of returning to sport following anterior cruciate ligament reconstruction surgery. *Phys Ther Sport*. 2008;9(1):9-15. doi:https://doi.org/10.1016/j.ptsp.2007.09.003
