# Supplementary material for: Biopsychosocial Factors Associated With Return to Preinjury Sport After ACL Injury Treated Without Reconstruction: NACOX Cohort Study 12-Month Follow-up
Source: Sports Health. 2022 May 27;15(2):176–84. doi: 10.1177/19417381221094780 (PMC9950991; doi:10.1177/19417381221094780)
Supplement: sj-docx-3-sph-10.1177_19417381221094780 – Supplemental material for Biopsychosocial Factors Associated With Return to Preinjury Sport After ACL Injury Treated Without Reconstruction: NACOX Cohort Study 12-Month Follow-up [file sj-docx-3-sph-10.1177_19417381221094780.docx]

**Appendix C**: **Statistical analysis**

*Univariable analysis*

We evaluated between-group differences in baseline data and the between-group differences in explanatory variables at 3, 6, and 12-month follow-up. Distributions were checked for normality and visual inspection of box plots was used to identify any outliers. As appropriate, independent samples *t* tests or Mann-Whitney *U* tests (for continuous variables), or chi-square tests with multiple Z tests, (for categorical variables), were used to establish if there were between-group differences. For independent samples *t* tests, Levene’s test for equality of variances was used. A p value of < 0.05 denoted statistical significance.

*Multivariable analysis*

We constructed the following three Generalised Estimating Equation (GEE) models:

- GEE model 1 - relationship between the explanatory variables measured at 3 months following injury, and return to sport at 12 months.
- GEE model 2 - relationship between the explanatory variables measured at 6 months following injury, and return to sport at 12 months.
- GEE model 3 - relationship between the explanatory variables measured at 12 months following injury, and return to sport at 12 months.

Age at ACL injury, GSES, and preinjury Tegner Activity Scale score were included as adjusting variables in each model.

Multicollinearity was checked for all independent variables and identified by tolerance values < 0.1.^1^ No interaction terms were included in the models. Goodness of fit, based on a generalisation of the likelihood, was assessed using the Quasi-likelihood under the Independence model Criterion (QIC) and the associated corrected statistic (QICC).^2^ Pearson residuals were used to identify outliers. We reviewed any outliers (Pearson residual >3) individually, and a decision to exclude outliers was made on a case-by-case basis.^5^

We estimated the target number of candidate explanatory variables prior to selecting the variables. The often-cited 10 cases per explanatory variable criterion and the rule-of-thumb N$\geq$ 50 + 8*m* (where *m* = number of independent variables)^5^ were used as guidelines. If we included 6 explanatory variables in each regression model, the minimum sample size was 60 participants, the target sample size was 98 participants [N = 50 + (8 × 6)]. For 5 explanatory variables, the minimum sample size was 50 participants and the target was 90 participants.

*Missing data*

Cases were excluded analysis by analysis for the univariable analyses and listwise for the GEE models. To assess the risk for response bias, we used a multivariate imputation using chained equations (MICE) approach^4,6^ to impute missing values for the explanatory variables at 3, 6 and 12 months (*mi impute chained* command). Then we re-calculated the odds ratios (*mi estimate:logistic* command) for each of the 3 multivariable models based on Rubin’s rules,^3,4^ and compared the odds ratios from the primary GEE models to the odds ratios calculated using the imputed dataset. The MICE approach was appropriate given the non-monotone (arbitrary) missing-value pattern. We assumed data were missing at random, and we checked the proportion of missing values and missing values patterns (*mi misstable* command).

We built conditional models using the independent variables: age at injury, sex, days between injury and baseline questionnaire, preinjury Tegner Activity Scale score, baseline SANE, baseline GSES score and return to preinjury sport. We varied the number of imputations from 20 to 50 and checked whether the results changed. The burn-in period for each chain was 10 iterations.

References

1. Hair JF, Black WC, Babin BJ, Anderson RE. *Multivariate Data Analysis*. 7. ed., Pearson new internat. ed. Pearson; 2014.

2. Hardin J, Hilbe J. Chapter 4: Residuals, Diagnostics, and Testing. In: *Generalized Estimating Equations*. 2nd ed. CRC Press; 2013.

3. Rubin DB, ed. *Multiple Imputation for Nonresponse in Surveys*. John Wiley & Sons, Inc.; 1987. doi:10.1002/9780470316696.fmatter

4. StataCorp. *Stata: Release 16 Statistical Software. Multiple-Imputation Reference Manual*. Release 16. StataCorp LLC; 2019.

5. Tabachnick BG, Fidell LS. Chapter 5: Multiple Regression. In: *Using Multivariate Statistics*. 6th edition. Pearson Education Limited; 2015.

6. White IR, Royston P, Wood AM. Multiple imputation using chained equations: Issues and guidance for practice. *Stat Med*. 2011;30(4):377-399. doi:https://doi.org/10.1002/sim.4067
