# Supplementary material for: Biopsychosocial Factors Associated With Return to Preinjury Sport After ACL Injury Treated Without Reconstruction: NACOX Cohort Study 12-Month Follow-up
Source: Sports Health. 2022 May 27;15(2):176–84. doi: 10.1177/19417381221094780 (PMC9950991; doi:10.1177/19417381221094780)
Supplement: sj-docx-4-sph-10.1177_19417381221094780 – Supplemental material for Biopsychosocial Factors Associated With Return to Preinjury Sport After ACL Injury Treated Without Reconstruction: NACOX Cohort Study 12-Month Follow-up [file sj-docx-4-sph-10.1177_19417381221094780.docx]

**Appendix D: Preinjury sports**

The table shows the preinjury sports reported by participants and the number of participants that reported each sport. Data are given for those who had and had not returned to preinjury sport at 12-month follow-up.

|  | **Number of participants** | | | |
| --- | --- | --- | --- | --- |
| **Preinjury Sport** | **Total**  *n* = 88 | **Returned** | **Not returned** | |
|  |  | *n* = 40 | | *n* = 48 |
| Soccer | 22 (25%) | 7 (17.5%) | | 15 (31.3%) |
| Floorball | 13 (14.8%) | 6 (15%) | | 7 (14.6%) |
| Running | 11 (12.5%) | 9 (22.5%) | | 2 (4.2%) |
| Strength training | 11 (12.5%) | 5 (12.5%) | | 6 (12.5%) |
| Aerobics | 7 (8.0%) | 0 | | 7 (14.6%) |
| Cycling/spinning | 3 (3.4%) | 2 (5%) | | 1 (2.1%) |
| Martial arts | 3 (3.4%) | 1 (2.5%) | | 2 (4.2%) |
| Walking | 3 (3.4%) | 2 (5%) | | 1 (2.1%) |
| Handball | 2 (2.3%) | 1 (2.5%) | | 1 (2.1%) |
| Ice hockey | 2 (2.3%) | 2 (5%) | | 0 |
| Basketball | 1 (1.1%) | 0 | | 1 (2.1%) |
| Dance | 1 (1.1%) | 0 | | 1 (2.1%) |
| Swimming | 1 (1.1%) | 1 (2.5%) | | 0 |
| Volleyball | 1 (1.1%) | 0 | | 1 (2.1%) |
| Other | 7 (8%) | 4 (10%) ^a^ | | 3 (6.3%) ^b^ |

^a^ Other sports: horse riding, motor sport and fitness; ^b^ Other sports: badminton, fitness and horse riding
