# Supplementary material for: Biopsychosocial Factors Associated With Return to Preinjury Sport After ACL Injury Treated Without Reconstruction: NACOX Cohort Study 12-Month Follow-up
Source: Sports Health. 2022 May 27;15(2):176–84. doi: 10.1177/19417381221094780 (PMC9950991; doi:10.1177/19417381221094780)
Supplement: sj-docx-5-sph-10.1177_19417381221094780 – Supplemental material for Biopsychosocial Factors Associated With Return to Preinjury Sport After ACL Injury Treated Without Reconstruction: NACOX Cohort Study 12-Month Follow-up [file sj-docx-5-sph-10.1177_19417381221094780.docx]

**Appendix E: Missing data**

The table shows the number of missing data points for each explanatory variable for those participants included in each of the 3 models.

| **Explanatory Variables** | **GEE model 1:**  **3 months**  (*n* = 85) | **GEE model 2:**  **6 months**  (*n* = 87) | **GEE model 3:**  **12 months**  (*n* = 88) |
| --- | --- | --- | --- |
|  | **Number of missing data points** | | |
| IKDC-SKF | 9 | 9 | 2 |
| ACL-QOL* | 13 | - | 4 |
| ACL-RSI | 14 | 11 | 4 |

*The ACL-QOL was not included in the 6-month follow-up questionnaire; *n* corresponds to number of participants included in the model.
