# Supplementary material for: Biopsychosocial Factors Associated With Return to Preinjury Sport After ACL Injury Treated Without Reconstruction: NACOX Cohort Study 12-Month Follow-up
Source: Sports Health. 2022 May 27;15(2):176–84. doi: 10.1177/19417381221094780 (PMC9950991; doi:10.1177/19417381221094780)
Supplement: sj-docx-6-sph-10.1177_19417381221094780 – Supplemental material for Biopsychosocial Factors Associated With Return to Preinjury Sport After ACL Injury Treated Without Reconstruction: NACOX Cohort Study 12-Month Follow-up [file sj-docx-6-sph-10.1177_19417381221094780.docx]

**Appendix F: Unadjusted Generalised Estimating Equation data**

The table shows biopsychosocial factors at 3 months, 6 months and 12 months, for participants who returned and did not return to preinjury sport at 12 months, with no adjusting variables.

| **Explanatory Variables** | **GEE model 1:**  **3 months**  (*n* = 71) | | **GEE model 2:**  **6 months**  (*n* = 76) | | | | **GEE model 3:**  **12 months**  (*n* = 83) | | | |
| --- | --- | --- | --- | --- | --- | --- | --- | --- | --- | --- |
|  | **OR (95% CI)** | **p** | **OR (95% CI)** | **p** | | | **OR (95% CI)** | | **p** | |
| IKDC-SKF | 1.0 (1.0 to 1.1) | 0.76 | 1.0 (1.0 to 1.1) | | 0.19 | 1.0 (1.0 to 1.1) | | 0.48 | |  |
| ACL-QOL* | 1.1 (0.6 to 2.0) | 0.85 | - | | - | 1.0 (0.5 to 1.9) | | 0.91 | |  |
| ACL-RSI | 1.3 (0.8 to 1.9) | 0.24 | 1.3 (0.9 to 1.8) | | 0.10 | 1.7 (1.1 to 2.7) | | 0.01 | |  |

*IKDC-SKF* subjective knee function, range 0-100; *ACL-QOL* knee-related quality of life, range 1-10; *ACL-RSI* psychological readiness, range 1-10; *OR*, odds ratio; *The ACL-QOL was not included in the 6-month follow-up questionnaire.
