# Supplementary material for: Biopsychosocial Factors Associated With Return to Preinjury Sport After ACL Injury Treated Without Reconstruction: NACOX Cohort Study 12-Month Follow-up
Source: Sports Health. 2022 May 27;15(2):176–84. doi: 10.1177/19417381221094780 (PMC9950991; doi:10.1177/19417381221094780)
Supplement: sj-docx-7-sph-10.1177_19417381221094780 – Supplemental material for Biopsychosocial Factors Associated With Return to Preinjury Sport After ACL Injury Treated Without Reconstruction: NACOX Cohort Study 12-Month Follow-up [file sj-docx-7-sph-10.1177_19417381221094780.docx]

**Appendix G: Adjusted Generalised Estimating Equation with imputed data**

The table shows biopsychosocial factors at 3 months, 6 months and 12 months, with adjusting variables, for participants who returned and did not return to preinjury sport at 12 months, using imputed data for the cases with missing data (50 imputations).

| **Explanatory Variables** | **GEE model 1:**  **3 months**  (*n* = 85) | | | **GEE model 2:**  **6 months**  (*n* = 87) | | | | | | | **GEE model 3:**  **12 months**  (*n* = 88) | | | | |
| --- | --- | --- | --- | --- | --- | --- | --- | --- | --- | --- | --- | --- | --- | --- | --- |
|  | **OR (95% CI)** | | **p** | **OR (95% CI)** | | | | **p** | | | **OR (95% CI)** | | **p** | | |
| IKDC-SKF | 1.0 (1.0 to 1.1) | | 0.62 | | 1.1 (1.0 to 1.1) | | | | 0.02 | | 1.0 (1.0 to 1.1) | | 0.35 | | |
| ACL-QOL* | 1.1 (0.6 to 2.0) | | 0.83 | | - | | | | - | | 0.9 (0.4 to 1.9) | | 0.70 | | |
| ACL-RSI | 1.3 (0.8 to 2.0) | | 0.24 | | 1.1 (0.8 to 1.6) | | | | 0.48 | | 1.9 (1.1 to 3.0) | | 0.01 | | |
| **Adjusting Variables** | |  | | | |  |  | | |  | |  | | |  |
| Age | 1.1 (1.0 to 1.2) | | 0.02 | | 1.1 (1.0 to 1.2) | | | | 0.01 | | 1.1 (1.0 to 1.2) | | | 0.03 | |
| Preinjury Tegner Activity Scale | 1.0 (0.8 to 1.3) | | 0.80 | | 1.0 (0.8 to 1.1) | | | | 0.88 | | 1.1 (0.8 to 1.3) | | | 0.65 | |
| GSES Baseline | 1.0 (0.9 to 1.1) | | 0.94 | | 1.0 (0.9 to 1.1) | | | | 0.90 | | 1.0 (0.8 to 1.1) | | | 0.55 | |

*IKDC-SKF* subjective knee function, range 0-100; *ACL-QOL* knee-related quality of life, range 1-10: *ACL-RSI* psychological readiness, range 1-10; *OR*, odds ratio; *GSES*, general self-efficacy scale; *The ACL-QOL was not included in the 6-month follow-up questionnaire.
